# Genetic contribution of catechol-O-methyltransferase variants in treatment outcome of low back pain: a prospective genetic association study

**DOI:** 10.1186/1471-2474-13-76

**Published:** 2012-05-21

**Authors:** Ahmad Omair, Benedicte Alexandra Lie, Olav Reikeras, Marit Holden, Jens Ivar Brox

**Affiliations:** 1 Department of Orthopaedics, Oslo University Hospital-Rikshospitalet, Oslo, Norway; 2 Department of Medical Genetics, University of Oslo and Oslo University Hospital-Ullevål, Oslo, Norway; 3 Norwegian Computing Center Blindern, Oslo, Norway; 4 Department of Orthopaedics, Oslo University Hospital-Rikshospitalet, Sognsvannsveien 20, 0027, Oslo, Norway

## Abstract

**Background:**

Treatment outcome of low back pain (LBP) is associated with inter-individual variations in pain relief and functional disability. Genetic variants of catechol-O-methyltransferase (*COMT*) gene have previously been shown to be associated with pain sensitivity and pain medication. This study examines the association between *COMT* polymorphisms and 7–11 year change in Oswestry Disability Index (ODI) and Visual Analog Score (VAS) for LBP as clinical outcome variables in patients treated with surgical instrumented lumbar fusion or cognitive intervention and exercise.

**Methods:**

93 unrelated patients with chronic LBP for duration of >1 year and lumbar disc degeneration (LDD) were treated with lumbar fusion (*N* = 60) or cognitive therapy and exercises (*N* = 33). Standardised questionnaires assessing the ODI, VAS LBP, psychological factors and use of analgesics, were answered by patients both at baseline and at 7–11 years follow-up. Four SNPs in the *COMT* gene were successfully genotyped. Single marker as well as haplotype association with change in ODI and VAS LBP, were analyzed using Haploview, linear regression and R-package Haplostats. P-values were not formally corrected for multiple testing as this was an explorative study.

**Results:**

Association analysis of individual SNPs adjusted for covariates revealed association of rs4633 and rs4680 with post treatment improvement in VAS LBP (*p* = 0.02, mean difference (*β)* = 13.5 and *p* = 0.02, *β* = 14.2 respectively). SNPs, rs4633 and rs4680 were found to be genotypically similar and in strong linkage disequilibrium (LD). A significant association was found with covariates, analgesics (*p* = 0.001, *β* = 18.6); anxiety and depression (*p* = 0.008, *β* = 15.4) and age (*p* = 0.03, mean difference per year (*β)* = 0.7) at follow-up. There was a tendency for better improvement among heterozygous patients compared to the homozygous. No association was observed for the analysis of the common haplotypes, these SNPs were situated on.

**Conclusions:**

Results suggest an influence of genetic variants of *COMT* gene in describing the variation in pain after treatment for low back pain. Replication in large samples with testing for other pain related genes is warranted.

## Background

Lumbar disc degeneration (LDD) represents a cause of the low back pain (LBP) [1]. A strong correlation between the severity of degeneration and LBP has not been established. Some studies have found a relationship between the radiological findings of LDD and LBP [2], while others indicate a high prevalence of abnormal radiological changes in asymptomatic individuals [3,4]. A substantial inter-individual variation among LDD patients with regard to the pain sensitivity and the requirement of post-operative analgesia has been recognized. Thirty five to 68 percent of the LBP susceptibility has been reported to be accounted for by the genetic factors [5].

Variation in certain genes encoding proteins involved in pain modulation, transduction, transmission, and conduction pathways (pain-genes), could be helpful in classifying the LDD patients experiencing varying degrees of pain. With regard to the variation in human pain phenotypes, associations have been reported with single nucleotide polymorphisms (SNPs) in genes coding for catechol-O-methyltransferase (COMT), opioid receptors (OPRM1, OPRD1), transient receptor potential (TRPV1, TRPA1), fatty acid amide hydrolase (FAAH) and α-subunit of voltage gated sodium channel (SCN9A)[6-11]. Among these, *COMT* gene variations (more than 30 SNPs) are the most studied in both human and experimental models.

COMT is an enzyme that mediates the O-methylation of certain catecholic pain pathway neurotransmitters including catecholamines (adrenaline and noradrenaline), dopamine, catecholestrogens and their hydroxylated metabolites [12-14], thereby eliminating their biological activity and toxicity. COMT is highly expressed in liver, kidney, brain, adrenal and lungs [15]. Although the exact mechanism is unclear, it has been proposed that it acts by effecting the adrenergic, noradrenergic and dopaminergic pain modulating and processing mechanisms [16,17].

The *COMT* gene is located on chromosome 22q11.2 [18]; with a 1.3 kb transcript producing the low affinity soluble *S-COMT* and a 1.5 kb transcript producing both soluble and high affinity membrane bound *MB-COMT*[15]. Polymorphisms of the *COMT* gene have been shown to contribute to the inter-individual variations in sensitivity, severity and chronicity of pain as well as its response to analgesics [8,19]. Pain intensity is also influenced by psychological factors and use of pain medication. Anxiety and depression are often associated with chronic pain [20].

The most studied *COMT* SNP (rs4680. *Val*158*Met*) is located in the coding region, where a non-synonymous *A* to *G* single nucleotide change causes replacement of valine (*Val*) to methionine (*Met*) at codon 158 in *MB-COMT* and 108 in *S-COMT*[21]. Met158 has lower thermo-stability and hence a decreased enzyme activity at normal body temperature as compared to the *Val*158 [22]. With regard to the genotypes, it has been shown that *Val*158 homozygous individuals have three-fourfold increased activity of COMT compared to the *Met*158 homozygotes, and an intermediate COMT activity with heterozygotes [23]. The COMT activity showed an inverse correlation with pain sensitivity. It has also been reported that in response to muscular pain, individuals homozygous for the 158*Met* allele possessed decreased activation and higher density of μ-opioid receptors in brain compared to 158*Val* homozygotes [24]. In another study involving cancer pain patients, it was reported that 158Val homozygotes required a 50% higher morphine dose than the 158*Met* homozygotes in [25].

An allele at another coding *COMT* polymorphism rs6267, also encodes a substantially less active enzyme but its prevalence is much lower [26]. In the non-coding region of the *COMT* gene, rs2075507 has been shown to impart a minor effect on the enzyme activity through altering mRNA expression [27].

A recent study found an association of a *COMT* SNP rs4633, as well as of a haplotype containing rs4633, rs4680, rs6269 and rs4818 with greater improvement in 1 year post-operative Oswestry Disability Index (ODI) score in patients who had fusion for persistent LBP [28].

On the basis of variation in experimental pain sensitivity; a study has identified a high pain sensitivity (HPS) or low COMT activity haplotype, a low pain sensitivity (LPS) or higher COMT activity haplotype, both including the similar amino acid sequence of *Val*158*Met* polymorphisms and differing in a synonymous SNP [8]. The APS/HPS diplotype causing the low COMT activity was also found to be associated with higher pre-operative pain scores and increased risk for post-operative pain [29].

In light of the broad spectrum of pain ratings in patients with LDD treated for LBP, we aimed to investigate the effect of the genetic variation in *COMT* gene on the pain and disability scores among patients treated with lumbar instrumental fusion or cognitive intervention and exercises.

## Methods

### Study design

This study is a prospective genetic association study. The cohort used was originally designed as a randomized control trial and the prospective design was used for obtaining data at follow up but results are given for the whole cohort with adjustment for the treatment received.

### Patient sample

124 unrelated Norwegian patients with chronic LBP and age ranging from 25–60 years, who were recruited from two randomized control trials (RCTs) published previously, were invited to participate. Results from the two RCTs showed no difference among groups randomized to surgical and cognitive intervention and exercises and the studies were merged at long term follow-up [30-33]. At baseline, each patient was examined by at least one spine surgeon and one specialist in physical and rehabilitation medicine. All patients underwent plain radiography of lumbar spine. The inclusion criteria were: age ≥ 25 years; LBP, duration >1 year despite of undergoing supervised non operative treatment; ODI score > 30 of 100 points; and disc degeneration at no more than two levels i.e. L4-L5 and/or L5-S1 as assessed by plain radiography. Exclusion criteria were: widespread myofascial pain; spinal stenosis with reduced walking distance and associated neurological signs; recurrent disc herniation or lateral recess stenosis with clinical signs of rediculopathy; inflammatory disease; previous spinal fracture; previous surgical fusion of spine; pelvic pain; generalized disc degeneration on plain radiographic assessment; ongoing serious somatic or psychiatric disease that could exclude treatment alternatives; registered medical abuse and reluctance in accepting either one or both treatment modalities of this study.

Ninety nine patients responded to the follow-up examination at mean 8.5 years (range 7–11 years). Ninety three patients were ethnically West European and hence 6 were excluded due to a non European ethnicity. Fifty one were randomized to lumbar instrumented fusion and 42 to cognitive intervention and exercises. Five patients randomized to fusion did not undergo surgery and 14 patients randomized to cognitive treatment had later undergone fusion. Demographics and pain characteristics of the 93 patients with LBP, according to the treatment received are given in Table 1. Among the 19 patients who did not receive the treatment they were randomized to, 7 were males and 12 were females, mean age was 53.5 years and ODI and VAS LBP change given as mean [SD] were – 22.6 [24.8] and – 26.3 [30.0] respectively.

**Table 1:** Demographics and pain characteristics of 93 patients with low back pain given as numbers or percentages or means [SD]

**Treatment**	**Lumbar fusion**	**Cognitive-exercises**
Number of patients	60	33
Gender (M/F)	20/40	15/18
Age at follow-up, years	52.3 [8.0]	51.1 [8.0]
Baseline ODI	46.0 [11.8]	40.6 [8.5]
Follow-up ODI	23.8 [18.6]	24.6 [15.9]
Change in ODI	- 22.2 [23.0]	−16 [15.4]
BaselineVAS LBP	63.4 [14.6]	60.6 [11.6]
Follow-up VAS LBP	35.6 [27.0]	41.8 [22.1]
Change in VAS LBP	- 27.8 [29.2]	−18.8 [23.2]
Percentage patients taking daily analgesics at follow-up	51	24
Percentage patients reported moderate to severe anxiety and depression at follow-up	31	35

The eligible patients were informed orally as well as in writing about the study procedures before consenting. The Regional Committee for Medical Research Ethics in Health Region South-East Norway approved the study. It was also recommended by Biobank register at the Norwegian Institute of Public Health and by patient’s ombudsman at Oslo University Hospital.

### Study interventions

The surgery was postero-lateral fusion with transpendicular screws at L4-L5 and /or L5-S1. Cognitive intervention and exercises were given by physiotherapists and a specialist in physical and rehabilitation medicine, and involved one week plus two weeks at outpatient facility interrupted by two weeks at home.

### Predictors and outcome variables

The clinical data regarding age, ethnicity, gender, and co-morbidity was obtained at baseline. At baseline and follow-up, these patients filled in standardised questionnaires for assessing the ODI and VAS (Visual Analog Scale) LBP scores. ODI comprised of 10 questions about pain and pain related disability, each having six verbal response alternatives. The sum of the response score is calculated and presented as percentage where 0% represents no pain and disability, and 100% represents the worst possible pain and disability [34].

Low back pain intensity was scored on three vertical visual analog scales, ranging from 0 (no pain) to 100 (worst pain imaginable). Patients scored their maximum pain, minimum pain and current pain at last week, respectively. The mean of these three measurements was calculated [35].

Information on the use of analgesics was reported by patients as four response alternatives: daily; weekly; monthly and less often [36]. Pain medication was dichotomized into using daily pain medication and less often.

Information on anxiety and depression was acquired through EuroQol questionnaire EQ-5D [37,38] and Hopkins Symptom Check List (HSCL-25) [39]. These variables were correlated (*r* = 0.7). In the present study we used the domain from EQ-5D, dichotomized into no anxiety and depression and moderate to severe anxiety and depression.

Clinical outcome variables used were the change in ODI and VAS LBP scores from baseline to 7–11 year follow-up (post treatment value – pre treatment value), that is the change for the whole cohort overtime and not the difference in change between treatment groups.

### Genotyping

Genomic DNA from 87 patients was extracted from 9 ml of venous blood by salting out method [40] and for the remaining 6 patients from 2 ml of saliva using a collection kit (DNA genotek, kanata, Ontario Canada). Based on the previously proposed association between genetic variations of catechol-O-methyltransferase (*COMT*) and difference in pain sensitivity, five SNPs (rs4633, rs4680, rs4818, rs6269 and rs2097603) were selected for genotyping [28]. Genotyping for these SNPs was performed by Sequenom^TM^ system, using matrix-assisted laser desorption/ionization time-of-flight (MALDI-TOF) mass spectrometry at the Centre for interactive genetics; Cigene, Norwegian University of Life Sciences (UMB) Aas.

### Statistical Analysis

Hardy-Weinberg equilibrium, pair wise linkage disequilibrium (LD), genotype success rate and minor allele frequency were calculated using the statistical programme Haploview version 4.2 [41]. The cut off value for divergence from Hardy-Weinberg equilibrium was (*p* ≥ 0.001). Inclusion of SNPs in this study was subject to the threshold for genotype success rate (GSR) set to be > 95%.

Linear regression analysis was performed for assessing the effect of each individual SNP on the ODI and VAS LBP change as dependent variables, along with controlling for covariates such as age, gender, fusion, analgesics and psychological factors. Normality was tested by histograms and Q-Q plots of residuals and was found to be acceptable for regression analysis.

Analysis was performed by using both recessive and additive genetic models. In the recessive model, we assumed that the change in post treatment pain scores was increased / decreased when having two copies of a specific allele compared to having only one or zero copies of the specific allele. Mean difference in pain score (*β*) between patients having two copies and those having one or zero copies was estimated in regression analysis. In the additive model, we assumed that the change in the post treatment pain scores was increased two fold by possessing two copies of the specific allele, compared to having a single copy. A *p*-value ≤0.05 was considered statistically significant. This was an explorative study and therefore the p-values were not formally corrected for multiple testing. The effect size for power 0.8 and significance level of 0.05 for rs4633 and rs4680 was 0.65 and a conservative estimate for smallest difference for VAS LBP change was calculated to be 17.4.

Association analysis between the common haplotypes of the SNPs with frequency > 0.02 and ODI and VAS LBP change, along with effect of covariates was performed on data from the whole cohort of 93 patients using the R package Haplostats [42]. The estimated frequencies and regression coefficients were computed by the function haplo.glm using an additive model by default. The reference haplotype was selected to be the most frequent haplotype as a baseline for linear regression by the software.

## Results

The genotype success rate was ≥ 97% for all SNPs (Table 2), except rs2097603 (71%), which was therefore excluded. No divergence from Hardy-Weinberg equilibrium was observed for any of the tested markers (Table 2). The minor allele frequencies observed for each SNP in our population were comparable to what has been reported for European Hap Map samples (Table 2).

**Table 2:** **Overview of the selected*COMT*SNPs**

SNP*	**Location**	HWE** ***p*-val**	**GSR %**†	**Alleles** ‡	**MAF**§ **cases**	**MAF reported**
rs4633	Exon 3	0.92	97.8	C/T	0.46	0.52
rs4680	Exon 4	1.0	100	G/A	0.46	0.52
rs4818	Exon 4	1.0	100	G/C	0.41	0.48
rs6269	Intron 2	0.91	100	G/A	0.42	0.49

* Single nucleotide polymorphism; ** Hardy-Weinberg equilibrium; † Genotype success rate;

‡ Minor/Major alleles; § Minor allele frequency.

### Single marker association analysis

In order to test an association of individual SNPs with the 7–11 years change in ODI and VAS LBP scores, a recessive genetic model was used in addition to the additive genetic model due to the restricted power of our sample. In the presence of age, gender and fusion as covariates, recessive model analysis of 93 patients (both surgical and cognitive-exercise) revealed associations between *COMT* polymorphisms rs4633 and rs4680 and post treatment reduction in pain (*p* = 0.05, *β* = 12.1 and p = 0.04, *β* = 12.7 respectively). These covariates were found to be non significant. No association was observed for the remaining two SNPs.

The association between rs4633 and rs4680 and reduction in pain was statistically significant (*p* = 0.02) after adjustment for consumption of analgesics, and anxiety and depression. These covariates were found to be significantly associated with reduction in pain (Table 3).

**Table 3:** Single marker association analysis of COMT SNPs and 7–11 years follow-up VAS LBP change in 93 patients, a recessive genetic model

	**rs4633**		**rs4680**	
	**Mean difference (*β*)† (95% CI)**	***p*value**	**Mean difference (*β*) (95% CI)**	***p*value**
SNP*§	13.5 (1.9, 25.0)	0.02	14.2 (2.7, 25.6)	0.02
Age	0.7 (0.05, 1.3)	0.04	0.7 (0.08, 1.4)	0.03
Gender	6.9 (−3.9, 17.7)	0.21	7.8 (−2.8, 18.5)	0.15
Fusion	11.82 (0.8, 22.8)	0.04	10.9 (0.1, 21.6)	0.05
Analgesics	18.4 (7.2, 29.6)	0.002	18.6 (7.5, 29.7)	0.001
Anxiety and depression	14.6 (3.2, 26.0)	0.01	15.4 (4.1, 26.7)	0.008

* Single nucleotide polymorphism, † mean difference in pain score (for age *β* is the mean difference in pain score per year); § Recessive genotype (rs4633: *T/T*; rs4680: *A/A*).

Reduction in pain was largest among patients who reported to take analgesics weekly or less often at follow-up and in patients who reported no anxiety and depression at follow-up. SNPs explained 3% of the variation and 11% with age, gender and fusion. When analgesics and anxiety and depression were entered into the model, the explained variance increased from 11 to 31%. No association was observed for any of the SNPs with ODI change, although the ODI change explained 60% of variance in pain change.

A sensitivity analysis performed according to intention to treat, revealed associations similar to the analysis based on treatment received. In the additive genetic model we observed no significant association between the tested markers and the follow-up ODI and VAS LBP change.

**Figure 1:**
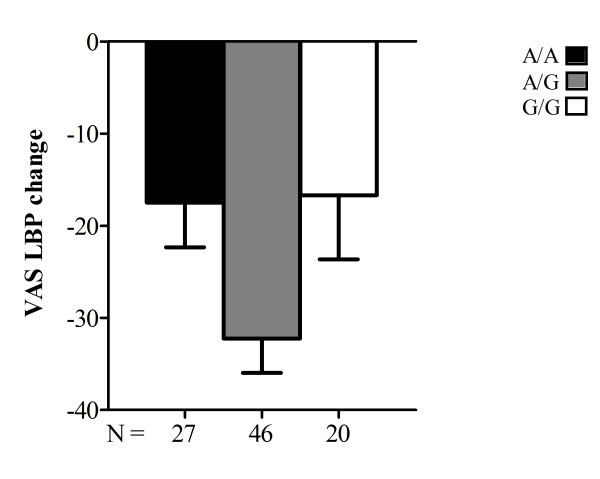
**Bar plot diagram of 93 patients for post treatment VAS LBP change.** Bar plot diagram of 93 patients for post treatment VAS LBP change (given as mean) in three genotypes (*A/A*, *A/G*, *G/G*) of rs4680. The error bar denotes the standard error of the mean; *N* denotes the number of patients for each genotype and the height of the filled rectangle boxes denotes the mean

**Figure 2:**
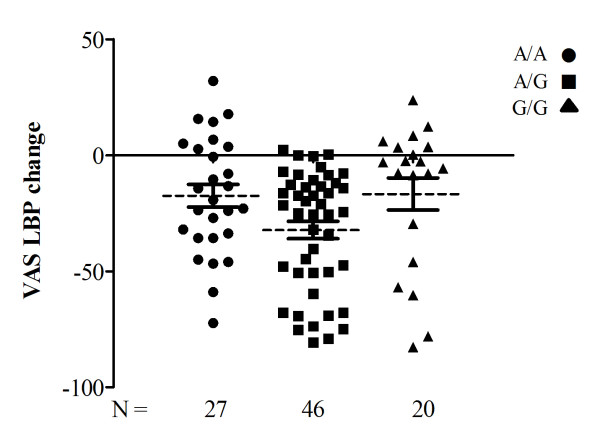
**Scatter plot diagram of 93 patients for post treatment VAS LBP change.** Scattergram of 93 patients for post treatment VAS LBP change in three genotypes (*A/A*, *A/G*, *G/G*) of rs4680. The dashed line represents the mean value and continuous lines denote standard error of mean

The two polymorphisms rs4680 and rs4633 were in strong linkage disequilibrium [LD] (*D'* = 1 and *r*^*2*^ = 1) and hence *A/A*, *A/G* and *G/G* of rs4680 corresponded to the *T/T*, *T/C* and *C/C* of rs4633. Among the total group of 93 patients, two patients lacked rs4633 data while none lacked results for rs4680. Therefore, as these two SNPs were perfectly correlated (*r*^*2*^ = 1), we chose to focus on rs4680 in further analysis. We observed that patients heterozygous for rs4680 alleles had a greater improvement in pain at long term follow-up (Figures 1 and 2).

The reduction in pain (given as mean [SD] of change in VAS LBP score) was largest among *A/G* heterozygotes (−32.2 [25.4]), followed by a nearly equal reduction among *A/A* homozygotes (−17.5 [25.3]) and *G/G* homozygotes (− 16.7 [31.0]) (Table 4).

**Table 4:** Characteristics of the patients (N = 93) after stratification on rs4680 genotypes (Mean [SD])

**Variable**	**A/A**	**A/G**	**G/G**
No of patients	27	46	20
Gender (M / F)	6 / 21	18 / 28	11 / 9
Age at follow-up	51.8 [8.3]	52.0 [8.2]	51.7 [7.3]
Fusion / Cognitive	19 / 8	30 / 16	11 / 9
Baseline ODI	43.7 [10.2]	46.1 [11.9]	39.9 [9.2]
Follow-up ODI	26.1 [17.4]	22.1 [19.7]	25.8 [18.1]
ODI change	- 17.6 [17.0]	- 24.0 [22.6]	- 14.1 [19.7]
Baseline VAS LBP	59.9 [14.1]	64.4 [13.4]	61.3 [13.6]
Follow-up VAS LBP	42.5 [18.3]	32.1 [26.6]	44.6 [29.0]
VAS LBP change	- 17.5 [25.3]	- 32.2 [25.4]	- 16.7 [31.0]
VAS LBP change [Median]	- 19.3	- 24.7	- 4.3

Associations for rs4633 and rs4680 with improvement in pain among the fused group were also statistically significant, both in the absence and when controlled for covariates.

### Haplotype association analysis

In view of the previously proposed associations of different *COMT* haplotypes with pain sensitivity and the fact that more haplotypes have shown associations compared to the individual SNPs, we constructed haplotypes. All four SNPs were in strong LD, and the three most common haplotypes accounted for more than 96.6% of all observed haplotypes (Table 5). No association was observed for any of the haplotypes, with reduction in pain and disability at long term.

**Table 5:** Observed haplotypes and their estimated frequencies

**rs4633**	**rs4680**	**rs4818**	**rs6269**	**Frequency %**
T	A	C	A	52.7
C	G	G	G	40.1
C	G	C	A	3.8

## Discussion

In the present study we found significant associations between the *COMT* polymorphisms rs4633 and rs4680 (*Val*158*Met*) and pain reduction at long term follow-up among fused and non-fused patients with chronic LBP. The polymorphism rs4633 was in complete linkage disequilibrium (LD) with rs4680 and hence showed similar results.

Dai et al were the first to test the association between *COMT* variants and surgical outcome with regard to pain and disability in 69 patients with lumbar fusion. They reported an association between improvement in ODI score and both a single marker (rs4633) and a *COMT* haplotype [28]. Our results are in line with Dai et al. with regard to the associations observed between success of treatment and rs4633 but do not replicate their findings, as our associations were with pain reduction and not with ODI. In addition we reported association for rs4680 as well, which was not found in the study by Dai et al.

Although the pain genes could only explain 3.0% of the variance in pain change, association remained significant after adjustment for covariates. The most important covariates were the reported use of pain medication and anxiety and depression at follow-up, as the explained variance increased from about 11% for the pain gene with age, gender and treatment to about 31% with addition of pain medication and anxiety and depression. Twice as many fused patients (51%) used analgesics daily at follow up, compared with the cognitive intervention and exercises (24%) and although the co linearity and correlation statistics was acceptable for performing multiple regression analysis, fusion was both negatively (with pain medication) and positively associated with change in pain (Table 3). The observed associations suggest that it seems unlikely that treatment responses can be predicted solely by the analysis of *COMT* gene polymorphisms and it may be recommended to assess the influence of candidate genes for both disc degeneration and pain in a larger cohort.

In the present study the largest improvement was observed for *A/G* heterozygous patients using the recessive genetic model. These results are contrary to the dose effect of the SNP and along with lack of association observed in the additive model, suggest that the effect is not increasing. Studies have shown that the three genotypes of the rs4680 influence the human experience of pain differently, with *Met/Met* homozygous patients being more pain sensitive compared to the *Val/Val* and those heterozygous possessed an intermediate pain sensitivity [24]. Increased enzyme activity is inversely related with the pain sensitivity and it has been reported that *Val/Val* homozygotes produce an effective enzyme and vice versa, while the heterozygotes express an intermediate COMT activity [14]. Lotsch et al. reported that carriers of *Val/Val* alleles were more sensitive to pain compared to the non carriers [43]. The present findings of a larger pain reduction in heterozygous individuals after stratification according to genotypes, does not fit with the already reported results. Frequency of *Val/Met* heterozygotes was much higher in our sample compared to *Val/Val* and *Met/Met* homozygotes. Due to a small sample size the findings could be false and by chance as we have not observed pain improvement in homozygous individuals which should have been in line with assumed functional effect of the enzyme. A case control study involving 61 Turkish fibromyalgia patients has previously reported a higher frequency of *Val/Met* heterozygotes [44] and also an association between individuals heterozygous for *COMT* gene polymorphisms rs464312 and rs6269 and pain sensitivity has previously been reported [10].

The association observed in the present study was with change in pain scores at long term follow-up, while Dai et al. have assessed the change at 1 year and in contrast to our findings Dai et al. reported a greater improvement with rs4633 homozygotes and an intermediate improvement with heterozygotes. In the present study change in ODI and VAS LBP were moderately correlated (*r*^*2*^ = 0.6) and therefore it is not unlikely to detect an association with one variable and not with the other. Contrary to Dai et al. who reported a significant association between a haplotype involving rs4633, rs4680, rs6269 and rs4818 and greater improvement in ODI score, our associations for similar haplotypes did not reach statistical significance neither for ODI nor for pain change. This is despite the fact that we have found association for two individual SNPs i.e. rs4680 and rs4633, which were also a part of this haplotype. Diatchenko L et al have also reported that as a single SNP, rs4680 was not associated with pain sensitivity despite possessing the same amino acid sequence as part of different haplotypes possessing different COMT activity and pain sensitivities [8]. These minor discrepancies could be due to population differences in LD and haplotype structures. The contrasting results make it difficult to establish the true genotypic effect. Nevertheless both these studies support the polymorphism as being associated. Genetic studies ideally require a large patient number that can tolerate the correction for multiple testing in order to avoid false positive results. Patient sample of the present study is larger as compared to Dai et al. and included fused and non-fused patients.

The mechanism of pain modulation is complex and many studies have either failed to show any associations between *COMT* variation and pain sensitivity [45,46], while other studies have found an association with pain sensitivity and variation in dosage requirement of morphine in cancer pain patients [25].

Inclusion of covariates in the analysis and homogeneity with regard to genetic make up and ethnicity are the strengths of this study. The distribution of different genotypes of rs4680 (*A/A*, *A/G* and *G/G*) was homogenous among both surgical and cognitive-exercise group (*p* = 0.6), and hence there was no evidence for selection bias on the basis of genetic make up among patients treated by the two different modules.

The conservative estimate for smallest difference for VAS LBP change was set at 17.4 in this study. Despite having limitations of sample size and being underpowered, this study gives suggestion towards the direction in which future research should head in examining the different clinical phenotypes related to patients with lumbar disc degeneration.

LBP is a major cause of disability among the population of the industrialized world, in turn leading to a socio-economic deterioration [47]. The pathogenesis and etiology of lumbar disc degeneration is complex regarding both degenerative process and patient’s symptomatology with regard to pain and disability. Radiologically assessed changes in the discs may or may not cause pain [48] and there is a visible variation in the patient outcomes after treatment. Thus, there is an indication and need for spine researchers to expand their knowledge at the molecular level, with regard to genetics of the degeneration of the lumbar discs and an inter-individual difference in pain sensitivity following the treatments.

Co-morbidity in patients with LBP has also been reported. Hagen et al, reported that pain was more often located to the whole spine (not just the lower back), legs and head, in patients with LBP as compared to the normal healthy controls [49]. Another study reported an increased prevalence of subjective health complaints in patients suffering from sciatica due to a herniated disc, compared to the normal healthy population [50]. These findings suggest a role of underlying genetic factors related to the pain sensitivity and perception that can predispose the patient to chronic pain, not just in the lower back but in other parts of the body as well. Previous studies have focussed on disc degeneration and genetics to reveal spine specific risk factors, while the focus on the role of pain genetics in the clinical outcome of LBP patients has been lacking.

## Conclusions

This study reports an association between variation in the pain gene *COMT* and reduction in pain in patients with LBP and lumbar disc degeneration at long term follow-up. Our findings suggest that genetic variants of *COMT* gene may contribute to describe the success of treatment, but that psychosocial factors might be more important. These results warrant replication in large samples with testing for other pain related genes, which in turn may help explain the inter-individual variation and contribute to more predictable treatment outcome.

## Abbreviations

COMT, Catechol-O-methyltransferase; LDD, Lumbar disc degeneration; LBP, Low back pain; SNPs, single-nucleotide polymorphisms; ODI, Oswestry Disability Index (ODI); VAS, Visual Analog Score.

## Competing interests

The authors declare that they have no competing interest.

## Authors’ contributions

AO, BL, OR and JIB contributed to the conception and design and conduction of the study. JIB recruited and examined the patients, provided funding and approvals. MH, AO and BL conducted the statistical analysis. AO drafted the manuscript and all authors critically revised the manuscript and approved the final version. All authors read and approved the final manuscript.

## Pre-publication history

The pre-publication history for this paper can be accessed here:

http://www.biomedcentral.com/1471-2474/13/76/prepub
